# Degeneration of Dopaminergic Neurons Due to Metabolic Alterations and Parkinson’s Disease

**DOI:** 10.3389/fnagi.2016.00065

**Published:** 2016-03-30

**Authors:** Juhyun Song, Jongpil Kim

**Affiliations:** Department of Biomedical Engineering, Dongguk UniversitySeoul, South Korea

**Keywords:** dopaminergic neurons, metabolic diseases, aging, neurodegenerative diseases, Parkinson’s disease (PD)

## Abstract

The rates of metabolic diseases, such as type 2 diabetes mellitus (T2DM), obesity, and cardiovascular disease (CVD), markedly increase with age. In recent years, studies have reported an association between metabolic changes and various pathophysiological mechanisms in the central nervous system (CNS) in patients with metabolic diseases. Oxidative stress and hyperglycemia in metabolic diseases lead to adverse neurophysiological phenomena, including neuronal loss, synaptic dysfunction, and improper insulin signaling, resulting in Parkinson’s disease (PD). In addition, several lines of evidence suggest that alterations of CNS environments by metabolic changes influence the dopamine neuronal loss, eventually affecting the pathogenesis of PD. Thus, we reviewed recent findings relating to degeneration of dopaminergic neurons during metabolic diseases. We highlight the fact that using a metabolic approach to manipulate degeneration of dopaminergic neurons can serve as a therapeutic strategy to attenuate pathology of PD.

## Introduction

Changing lifestyles means that many older people suffer from metabolic syndromes, including type 2 diabetes (T2DM), obesity, and cardiovascular disease (CVD; Luchsinger, 2010). Aging is accompanied by a variety of physiological changes, such as mitochondrial dysfunction, inflammation and a decline in insulin sensitivity (Dillin et al., 2002; Lee et al., 2003; Short et al., 2005; Riera and Dillin, 2015), which finally lead to age-related diseases. According to a recent survey, the prevalence of metabolic diseases increases by approximately 46.7% among people older than 60 years compared to younger individuals (Sepúlveda and Murray, 2014). Furthermore, this prevalence increases by over 50% in people aged over 65 years (Aguilar et al., 2015). Consequently, as the global population ages there will be a higher prevalence of CVD, T2DM and obesity (Kalyani et al., 2014; Sepúlveda and Murray, 2014; Hinnouho et al., 2015). Moreover, several studies have found that metabolic diseases are significantly linked to neurodegenerative diseases such as Alzheimer’s disease (AD) and Parkinson’s disease (PD; Anthony et al., 2006; Tezapsidis et al., 2009; Ahtiluoto et al., 2010; Lara et al., 2013; Ninomiya, 2014). Patients with neurodegenerative diseases like PD commonly suffer motor disturbance due to the degeneration or loss of dopaminergic neurons (Michel et al., 2013; Kalia and Lang, 2015). In fact, one clinical research study demonstrated that dopaminergic neuron activity strongly influences metabolic alterations in humans (Brunerova et al., 2013). Thus, the loss of dopaminergic neurons and degeneration should be an area of research focus to identify a therapeutic solution for metabolic change-induced PD. Here, we review recent studies and provide new insight on the link between metabolic diseases and the degeneration of dopaminergic neurons, which consequently leads to PD.

## The Association Between Metabolic Diseases and PD

Among metabolic disorders, T2DM is associated with neurodegenerative diseases, including diabetic neuropathy and Alzheimer’s disease (Wild et al., 2004; Boulton et al., 2005). In addition, recent studies reported a positive correlation between diabetes and dementia risk (Cheng et al., 2012; Gudala et al., 2013; Vagelatos and Eslick, 2013). According to many epidemiological studies, the impairment of insulin action in people with T2DM, could lead to brain dysfunction (Boulton et al., 2005; Corti et al., 2011), such as cognitive decline, compared to people not suffering from the disorder (Lu and Hu, 2012). In particular, insulin resistance is thought to promote chronic hyperglycemia (Ahtiluoto et al., 2010), and leads to cognitive impairment and vascular dementia by promoting the secretion of proinflammatory cytokines (Ninomiya, 2014). Decreased insulin receptors in the PD brain is related to neuronal loss in the substantia nigra (Moroo et al., 1994). In fact, one clinical study suggested that diabetes results in Parkinsonian signs such as postural reflex and gait impairment (Arvanitakis et al., 2007). Furthermore, obesity is considered the main cause of hypothalamic inflammation (Tang et al., 2015). Excessive fat accumulation induces inflammatory responses by promoting proinflammatory cytokine production in the central nervous system (CNS; Velloso et al., 2015). There is a strong relationship between high levels of adipokines, generated by adipose tissues (Buchman et al., 2005), and cognitive decline, and the former can be used as a predictor of the latter (Zeki Al Hazzouri et al., 2013). Some studies indicated a remarkable effect of leptin, as an adipokine, on memory loss in obese animal models (Bigalke et al., 2011; Theodoropoulou et al., 2012). Another study demonstrated that leptin improves cognitive function in mice that showed motor dysfunction and memory decline (Farr et al., 2006). Furthermore, clinical studies report that patients with metabolic disorder also show decreased cognitive function (Vieira et al., 2011; Levin et al., 2014). Studies on PD show significantly decreased heart function in patients (Rodríguez et al., 1996; Kallio et al., 2000). The association between cardiovascular autonomic dysfunction and PD has been further highlighted by recent evidence (Cilia et al., 2015). Also, high blood pressure considered an important risk factor in CVD increases the prevalence of dementia (Qiu et al., 2005). Many evidences indicate that hypertension and blood pressure are strongly associated with neurotoxicity in the brain (Langbaum et al., 2012; Toledo et al., 2012; Rodrigue et al., 2013). Taken together, metabolic diseases including T2DM, obesity, and CVD are linked to the onset and development of PD. Considering connected consequences between these disorders, we suggest that investigation for common issues need to improve multiple pathologies in metabolic change-induced PD.

## Degeneration of Dopaminergic Neurons and PD

Dopamine is primarily regarded as a neurotransmitter that controls the brain reward system (Blum et al., 2000). Midbrain dopaminergic neurons in brain areas, such as the substantia nigra pars compacta, regulate movement activation and cognitive functions by binding dopamine D1 and D2 receptors (Palmiter, 2007, 2008; Kravitz et al., 2010; Tritsch and Sabatini, 2012; Calabresi et al., 2014). *In vivo* pharmacological studies have suggested that the blockade of dopamine receptors in the dorsolateral prefrontal cortex and dorsal striatum, reduces cognitive functions (Landau et al., 2009; Cools, 2011; Li and Mei, 1994; Sawaguchi and Goldman-Rakic, 1994). Loss of dopaminergic neurons triggers a deregulation of motor symptoms such as rigidity and postural instability (Rodriguez-Oroz et al., 2009; Masoud et al., 2015). PD is characterized by the progressive loss of dopaminergic neurons of the substantia nigra pars compacta that project to the striatum (Michel et al., 2013; Kalia and Lang, 2015). Imaging studies in PD patients have reported a positive correlation between cognitive deficits, and the reduction of dopamine levels in the frontostriatal circuit (Brück et al., 2001, 2005; Sawamoto et al., 2008; Jokinen et al., 2009). Postmortem studies in PD patients have shown the loss of cholinergic (Zweig et al., 1989), and dopaminergic neurons (Karachi et al., 2010) which are crucial in the progression of PD. Moreover, one study found that cognitive decline in PD is strongly associated with the disruption of dopamine signaling in the prefrontal cortex (Narayanan et al., 2013). The pathology of PD is strongly linked to the death of dopaminergic neurons and the presence of Lewy bodies (Chase et al., 1998). Another study also reported a relationship between dopamine neuron degeneration and cognitive impairments (Bromberg-Martin et al., 2010). The intracellular mechanisms of dopaminergic neurons, and the environmental conditions around them, contribute to the progress of familial and sporadic PD forms (Dauer and Przedborski, 2003; Rodriguez et al., 2014; Ryan et al., 2015). The cellular mechanisms related to dopaminergic neuron degeneration are associated with increased sensitivity to mitochondrial dysfunction (Jeon et al., 1995; Lotharius et al., 1999; Kann and Kovács, 2007) and changes in protein degradation (Mosharov et al., 2009; Yacoubian and Standaert, 2009; Dehay et al., 2015). These researches indicated that the change of mitochondria energy metabolism caused by metabolic changes influences the expression of PD related proteins such as LRRK2, α-Syn, PINK-1, UCH-L1, DJ-1. Recent studies found a number of causes for dopaminergic neuron degeneration such as oxidative stress (Segura-Aguilar et al., 2014), aging (Ba and Martin, 2015) and neuroinflammation (Ryan et al., 2015). Based on these studies, decreased dopamine transporters in dopaminergic neuron could be used as the indicator to diagnose PD, in that the level of dopamine decisively affects the pathology progress of PD. The damage to dopaminergic neurons is mediated by the apoptosis signal, p53, which was found using an *in vitro* PD model (Li et al., 2016). Based on previous evidence, the degeneration of dopaminergic neurons is an important issue in the progression of PD, and its mechanisms should be studied to identify therapeutic solutions using a variety of approaches.

## Degeneration of Dopaminergic Neurons During T2DM

### Insulin Resistance and Dopaminergic Neurons

The brain, as an energy expensive organ, consumes 20% of the oxygen and glucose in the body (Zlokovic, 2011). Insulin interacts with the blood brain barrier (BBB) to initiate signaling pathways within the brain (Kastin and Akerstrom, 2001; Kondo et al., 2004; Ueki et al., 2004; Banks et al., 2012). Appropriate insulin levels are necessary to maintain sufficient glucose transport to the brain (Reagan et al., 2001; Bingham et al., 2002; McNay et al., 2010). Insulin action in the brain has been reported to modulate neuronal survival, synaptic function, cognitive function and neuronal circuit formation (Cholerton et al., 2011; Banks et al., 2012). The hormone inhibits neuronal damage resulting from glucose–oxygen deprivation (Sun et al., 2010), excitotoxicity (Kim and Han, 2005) and oxidative stress (Ribeiro et al., 2014). Insulin receptors have been found in midbrain dopaminergic neurons (Figlewicz et al., 2003). It increases dopaminergic transporter activity and enhances the clearance of dopamine from the synapse (Davis et al., 2011). Insulin action influences the function of the dopamine transporter, which is a key regulator of dopamine neurotransmission (Speed et al., 2011) and is important for cognitive ability (Figlewicz and Benoit, 2009; Figlewicz and Sipols, 2010). In addition, insulin signaling in dopaminergic neurons plays a crucial role in energy balance and the reward system (Khanh et al., 2014). Impaired insulin signaling aggravates brain dysfunction related to dopamine homeostasis (Carvelli et al., 2002; Garcia et al., 2005; Owens et al., 2005; Wei et al., 2007). Previous researches demonstrated that brain insulin resistance controls both the reward system and dopamine system (Luo et al., 1999; Anthony et al., 2006). Moreover, insulin-like growth factor (IGF-1) receptors within the substantia nigra (Dunn and Castro, 1980), induce insulin signaling and stimulate the activation of the phosphatidylinositol 3-kinase/AKT/glycogen synthase kinase-3 beta and mitogen-activated protein kinase pathways (Avila-Gomez et al., 2010) that modulate cell survival. Several studies indicated that IGF-1 receptors could rescue dopaminergic neurons from apoptosis (Cheng et al., 2011; Zawada et al., 1996; Wang et al., 2010). Akt signaling related to insulin signaling (Hage Hassan et al., 2015; Sadi et al., 2015; Xu et al., 2015) is markedly associated with the regulation of dopaminergic signaling (Beaulieu et al., 2007, 2009) and dopaminergic homeostasis (Hanada et al., 2004; Garcia et al., 2005; Williams et al., 2007). Notably, clinical research studies reported that a human genetic variant of the Akt insulin signaling pathway, found in patients, dysregulated the dopamine system (Emamian et al., 2004; Ikeda et al., 2006). In *in vivo* studies, genetic modification of Akt results in the dysregulation of dopaminergic neuron homeostasis (Siuta et al., 2010). Another study showed that a decrease in plasma insulin levels in rats, results in impaired insulin action on dopaminergic neurons (Patterson et al., 1998). Also, the ablation of insulin receptors in dopaminergic neurons leads to intercellular damage to the neurons (Könner et al., 2011), while insulin administration to rats showed an increase in dopamine transporter protein (Figlewicz et al., 1994). Moreover, insulin resistance in the brain changes dopamine turnover and results in movement disorders (Kleinridders et al., 2015). Insulin resistance caused by a high-fat diet exacerbates dopaminergic degeneration in mice (Bousquet et al., 2012). Clinical studies found increased insulin resistance and decreased insulin concentrations in the cerebrospinal fluid of patients with neurodegenerative disorders (Bomfim et al., 2012; Talbot et al., 2012). Some surveys reported an increased PD risk in patients with T2DM, accompanied by insulin resistance (Hu et al., 2007; Sun et al., 2012). One study found that Daf-2/Daf-16 insulin-like signaling pathway is important in mechanisms connecting PD and diabetes such as alpha-synuclein pathology of PD under high glucose conditions (Fatima et al., 2014). Recent research, using *in vitro* and *in vivo* models, demonstrated that appropriate insulin levels could protect dopaminergic neuronal damage in the substantia nigra during PD (Pang et al., 2016). Recently, drugs to treat insulin resistance demonstrated protective effects in PD (Aviles-Olmos et al., 2013a,b). Overall, the role of insulin signaling on dopaminergic neurons in PD, in a background of T2DM-related insulin resistance, should be investigated to understand the underlying mechanisms.

### Inflammation Caused by Hyperglycemia and Dopaminergic Neurons

Several studies suggest that neuronal loss in PD is associated with chronic neuroinflammation related to microglia and immune cells in the brain (Barcia et al., 2003; Perry, 2012). Clinical studies observed higher levels of pro-inflammatory factors such as interleukin (IL)-1beta, IL-2, tumor necrosis factor (TNF)-alpha, interferon (IFN)-gamma, and cluster of differentiation (CD)4+ and CD8+ T lymphocytes in PD brains (Dobbs et al., 1999; Hisanaga et al., 2001; Reale et al., 2009). Microglial activation in the substantia nigra (Lawson et al., 1990) was commonly observed in PD patients (Doorn et al., 2014a), and in PD animal models (Doorn et al., 2014b; Pisanu et al., 2014; Stott and Barker, 2014). In oxidative stress caused by PD, microglia promote an inflammatory state contributing to dopaminergic neuronal degeneration (McGeer and McGeer, 2008; Frank-Cannon et al., 2009; Burbulla et al., 2010). According to these evidences, the activation of M2 type microglia which secrets anti-inflammatory cytokines such as transforming growth factor (TGF)-beta and IL-10 improves the pathology of PD. The production of pro-inflammatory cytokines by inflammation leads to the apoptosis of dopaminergic neuron. Impaired glucose tolerance was observed in 80% of PD cases (Sandyk, 1993; Hu et al., 2007). The high glucose condition in T2DM triggers oxidative stress and dopaminergic neuronal death (Giaccari et al., 2009; Zhang et al., 2011; Renaud et al., 2014; Yoon and Oh, 2015). Dopaminergic neurons in the brain deteriorate due to increased production of reactive oxygen species (ROS) under high glucose stress (Pearce et al., 1997; Brownlee, 2001; Cui et al., 2012). These researches indicated that insulin level and glucose dysregulation could influence dopaminergic neuron’s degeneration. Clinical research found that dopaminergic neurons were damaged through apoptosis signaling pathways, such as p53 (Tatton, 2000; Hartmann and Hirsch, 2001; Nair, 2006), in PD patients, and an increase in activated microglia was observed in the basal ganglia of PD patients (Ouchi et al., 2005). One study found that long term incubation under high glucose concentrations increased depolarization-induced dopamine release (Koshimura et al., 2003). In addition, studies also found a correlation between blood glucose levels and cerebrospinal fluid concentrations of dopamine metabolites (Umhau et al., 2003). Detrimental inflammatory conditions, resulting from high glucose concentrations, have been reported to induce degeneration and cell death of dopaminergic neurons in PD animal models (Hu et al., 2007; Cai, 2009; Morris et al., 2010; Machado et al., 2011; Yoon and Oh, 2015). In summary, given that metabolic inflammation exacerbates dopaminergic neuronal damage in response to T2DM (Wang et al., 2014), the inflammatory environment caused by T2DM should be controlled to attenuate dopaminergic neuronal degeneration in PD.

## Degeneration of Dopaminergic Neurons in Obesity

Improper functioning of dopaminergic neurons in obesity is an important issue (Stice et al., 2008). Reduced dopaminergic receptor 2 expression in obesity, has been reported (Stice et al., 2008, 2010). Another study found low expression levels of dopaminergic receptor 2 in obese subjects compared to non-obese controls (Wang et al., 2011). Dopamine D2 receptor in the striatum was markedly lower in obese rats compared to lean control rats (Hamdi et al., 1992). Moreover, one study showed that dopamine transporter levels negatively correlated with body mass index (BMI), suggesting that the dopamine system regulates BMI (Chen et al., 2008). Recent studies indicated that the improvement of dopamine function could be key to the treatment of obese subjects (Avena et al., 2013a,b; Blum et al., 2014; Curtis and Davis, 2014). The increase in dopaminergic neuron receptors D1 and D2 reduces body fat, serum levels of free fatty acid, glucose and insulin (Cincotta et al., 1997; Conti et al., 2001). Freeman et al. (2001) investigated the effect of glucose in dopamine neuronal activity and found that a change in activity is influenced by caloric intake. One study demonstrated that the dopamine system is sensitive to alterations in energy metabolism (van der Plasse et al., 2015). Dopaminergic neuron D2 receptor levels are inversely associated with the BMI of obese subjects (Volkow et al., 2009; Michaelides et al., 2012). According to some findings, obesity associated genes control the activity of midbrain dopaminergic circuitry (Hess et al., 2013). Dopaminergic neurons in the midbrain are reported to play a crucial role in controlling food seeking behavior (Salamone et al., 1997; Roitman et al., 2004; van Zessen et al., 2012), by affecting the metabolic state and some hormones (van Zessen et al., 2012). An *in vivo* study using leptin-null mice (leptin is a key molecule, which links metabolic information to reward signaling) showed that feeding behavior is influenced by dopamine levels (Szczypka et al., 2000). Recent research found a loss of dopaminergic neurons due to metabolic dysregulation in an animal model of obesity, under a chronic high fat diet (Khang et al., 2015). Therefore, further studies on dopaminergic neuronal degeneration in obesity may be crucial to alleviate obesity-induced neuropathophysiology.

## Degeneration of Dopaminergic Neurons in CVD

CVD is generally accompanied by hypertension and atherosclerosis due to the accumulation of atheromatous plaques in coronary arteries, and is one of the main causes of death worldwide (Braunwald, 1997). Some studies show that cardiac failure is involved during the onset and prevalence of PD (Zesiewicz et al., 2004; Ziemssen and Reichmann, 2007, 2010). Based on clinical research, approximately 35% of PD patients show cardiac denervations (Goldstein, 2006; Goldstein et al., 2012; Martorell-Riera et al., 2014). Recent results support the relationship between mitofusin 2, as a functional element in heart mitophagy, and PTEN-induced putative kinase 1 (PINK1), as a PD-related factor (Chen and Dorn, 2013). Cardiac sympathetic denervation in PD is central to understanding the relationship between CVD and dopaminergic neurons (Goldstein et al., 2007). Dopamine plays a beneficial role in hypertension patients (Lyzogub et al., 2014). Low dose dopamine activates dopaminergic receptors, and ameliorates vasodilatation of the coronary and cerebral vessels (Liang et al., 2008; Chen et al., 2013). Cardiac sympathetic denervation is significantly related to the loss of nigrostriatal dopaminergic neurons (Goldstein et al., 2008, 2011, 2012). PD is associated with decreased myocardial innervations and increased cardiac sympathetic denervation (Orimo et al., 2002; Amino et al., 2005). Several studies have reported the presence of the renin angiotensin system (RAS) in basal ganglia, and high concentrations of angiotensin converting enzyme have been found in the striatum and substantia nigra of mammals (Chai et al., 1987; Allen et al., 1992). An interaction between dopaminergic neurons and angiotensin II receptors were demonstrated by several studies on the regulation of cardiovascular function (Zeng et al., 2006; Khan et al., 2008; Gildea, 2009). Recent findings show abnormal interactions between dopaminergic neurons and angiotensin II, affecting both neurodegenerative diseases and hypertension (Gildea, 2009; Li et al., 2008). Chronic inhibition of RAS leads to increased dopaminergic neuronal function (Jenkins et al., 1997, 1999; Reardon et al., 2000; Villar-Cheda et al., 2010). RAS hyperactivity is related to dopaminergic neuron degeneration in PD, and involves oxidative stress and neuroinflammation (Villar-Cheda et al., 2010). Collectively, understanding the mechanisms connecting dopaminergic neuron degeneration and CVD, is a fundamental issue to improve PD pathologies, as dopaminergic neurons are involved in heart function by modulating the RAS system and controlling cardiac inflammation.

## Conclusion

**Figure 1:**
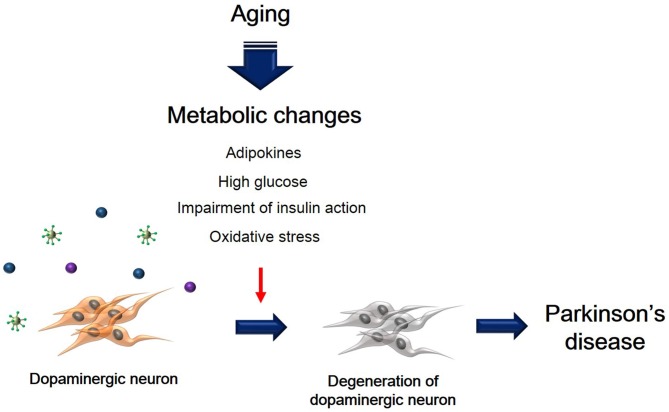
**The schematic image regarding dopaminergic neuron’s degeneration caused by metabolic changes.** This image shows that degeneration of dopaminergic neuron was accelerated by aging-induced metabolic changes including high glucose, insulin action’s impairment, oxidative stress, and adipokines.

Metabolic changes due to aging could affect multiple neuropathological mechanisms, and may also contribute to the progression and onset of PD (Figure 1). Insulin resistance and the hyperglycemia condition caused by T2DM lead to damage of dopaminergic neurons via several signaling pathways. In addition, inflammation, adipokine dysfunction and inappropriate energy metabolism by obesity, trigger the loss and degeneration of dopaminergic neurons. Heart failure related to RAS hyperactivity and cardiac inflammation cause the degeneration of dopaminergic neurons. Taken together, metabolic alterations could affect dopaminergic neuronal loss and degeneration, leading to PD neuropathology including motor disturbance and cognitive decline. Hence, we suggest that manipulating dopaminergic neuron degeneration by targeting metabolic pathways propose therapeutic approaches to ameliorate metabolic effects in PD.

## Author Contributions

JS wrote the preliminary draft and revised details of the manuscript. JK revised all manuscript in detail.

## Conflict of Interest Statement

The authors declare that the research was conducted in the absence of any commercial or financial relationships that could be construed as a potential conflict of interest.
